# Possible contribution of sialic acid to the enhanced tumor targeting efficiency of nanoparticles engineered with doxorubicin

**DOI:** 10.1038/s41598-020-76778-9

**Published:** 2020-11-12

**Authors:** Song Yi Lee, Suyeong Nam, Ja Seong Koo, Sungyun Kim, Mingyu Yang, Da In Jeong, ChaeRim Hwang, JiHye Park, Hyun-Jong Cho

**Affiliations:** 1 College of Pharmacy, Kangwon National University, Chuncheon, Gangwon 24341 Republic of Korea; 2 Kangwon Institute of Inclusive Technology, Kangwon National University, Chuncheon, Gangwon 24341 Republic of Korea

**Keywords:** Biological techniques, Biotechnology, Cancer, Medical research, Materials science, Nanoscience and technology

## Abstract

Doxorubicin (DOX)-engineered poly(lactic-co-glycolic acid) (PLGA) nanoparticles (NPs) including phloretin (PHL) were designed and the feasible contribution of sialic acid (SA) to the improved tumor targeting and penetration capabilities was elucidated in lung adenocarcinoma models. DOX has been clinically used as liposomal formulations after its introduction to the inner side of vehicles, however DOX is anchored in the outer surface of PLGA NPs for improved tumor penetration by interactions with SA in this study. DOX (positively charged at physiological pH) was adsorbed onto the negatively charged PLGA NPs via electrostatic interactions and consequent binding of SA (negatively charged at physiological pH) to DOX located in NPs was also elucidated. DOX layer in DOX@PLGA NPs rendered improved endocytosis and partial contribution of SA (expressed in cancer cells) to that endocytosis was demonstrated. DOX@PLGA/PHL NPs provided enhanced antiproliferation potentials in A549 cells rather than single agent (DOX or PHL)-installed NPs. In addition, DOX-SA interactions seemed to play critical roles in tumor infiltration and accumulation of DOX@PLGA NPs in A549 tumor-xenografted mouse model. All these findings support the novel use of DOX which is used for the surface engineering of NPs for improved tumor targeting and penetration.

There have been a lot of advances in the development of nanomedicines for cancer imaging and therapy^1–9^. Accurate tumor-targeted drug delivery is very crucial for elevating anticancer efficacies and minimizing unwanted toxicities in cancer therapy. Around tumor tissues, leaky vascular structures due to poor differentiation and immature lymphatic drainage may allow for the extravasation of macromolecules or nanomaterials^10,11^. Nanostructures with hundreds of nanometers can be easily transferred to tumor tissue via an enhanced permeability and retention (EPR) effect which is known as a passive targeting approach^12,13^. Based on the passive tumor targeting method, several nanomedicines (*i.e.,* Genexol-PM, Abraxane, and Doxil) got approval for clinical application in cancer therapy^14^. However, there are some mixed responses against the usefulness of EPR effect in clinics mainly due to differences in the anatomical structures of human and experimental animals^15^. Therefore, active tumor targeting methods (*i.e.,* ligand-receptor interactions) have been devised as one of more elaborated drug delivery approaches^16^.

These passive and active targeting methodologies may be governed by physicochemical properties of materials and nanostructures as well as the biological aspects of tumor tissues^16^. Various kinds of materials have been screened for their application to the design of tumor-targeted nanosystems^17–24^. Amid so many types of materials, poly(lactic-co-glycolic acid) (PLGA) has been widely used due to its biocompatibility and biodegradability^3,25^. PLGA can be degraded into lactic acid (LA) and glycolic acid (GA), then participating in the metabolic pathway in the body^25,26^. PLGA has been approved by the European Medicine Agency and the United States Food and Drug Administration for injection dosage forms^27^. Along with safety factors, PLGA-based nanostructures have been conveniently engineered for maximizing tumor targeting capability and drug delivery efficiency^3,25^. Small molecules, peptides, proteins, and nucleic acids can be chemically linked or physically adsorbed to the outer surface of PLGA-based NPs to provide tumor targeting and therapeutic capabilities^28–31^. In spite of hydrophobic nature of PLGA, hydrophilic or hydrophobic drug cargos can be coated to the outer layer of PLGA NPs or loaded to the internal space of PLGA NPs^3^.

In this investigation, doxorubicin HCl (DOX)-engineered PLGA NPs (DOX@PLGA NPs) containing phloretin (PHL) were designed for the enhancement of tumor targeting and penetrating potentials. Although the application of DOX-hydroxyapatite-PLGA nanocomposites to the treatment of cancer has been reported^32^, the targeting and penetrating capabilities of DOX to tumor tissues were not investigated yet. The pK_a_ value of primary amine group included in DOX is known as 8.2–9.9^33–35^, therefore it will behave as a cationic molecule at acidic and neutral pH. In addition, among so many molecules overexpressed in cancer cells, sialic acid (SA) has been used as one of representative tumor targets which can be associated with ligands (*i.e.,* phenylboronic acid)^21,36,37^. Upregulation of terminal SA structure has been regarded as a hallmark of cancer and it may reduce the adhesion of tumor cells to the extracellular matrix and block detection by alternative complement activation pathway^38,39^. The pK_a_ value of carboxylic acid group in SA is 2.6 and it may exhibit an negative charge at physiologically pH^40^. Therefore, electrostatic interactions between positively charged DOX (located in the surface of DOX@PLGA NPs) and negatively charged SA (present in cancer cells) may contribute to the selective endocytosis of DOX-engineered PLGA NPs in SA-overexpressed cancer cells. Contrary to the incorporated DOX molecules in the liposomal structures (*i.e.,* Doxil) which are clinically available, externally attached DOX onto NPs is expected to provide tumor targeting and penetrating capabilities as well as chemotherapeutic efficacies in tumor tissues. Moreover, PHL is believed to amplify the anticancer potentials by the inhibition of glucose transport in cancer cells. To the best of our knowledge, dual roles (association with SA for tumor targeting and combination anticancer effects) of DOX were first introduced to chemotherapy of cancers in this study. Optimization of the distribution of DOX in the surface of PLGA NPs may enhance anticancer efficacies by tumor targeting and its own chemotherapeutic natures without severe toxicities following intravenous administration. Physicochemical and biological functions of DOX-engineered PLGA NPs including PHL will be systemically assessed in this study.

## Results and discussion

### Fabrication and physicochemical properties of NPs

**Figure 1:**
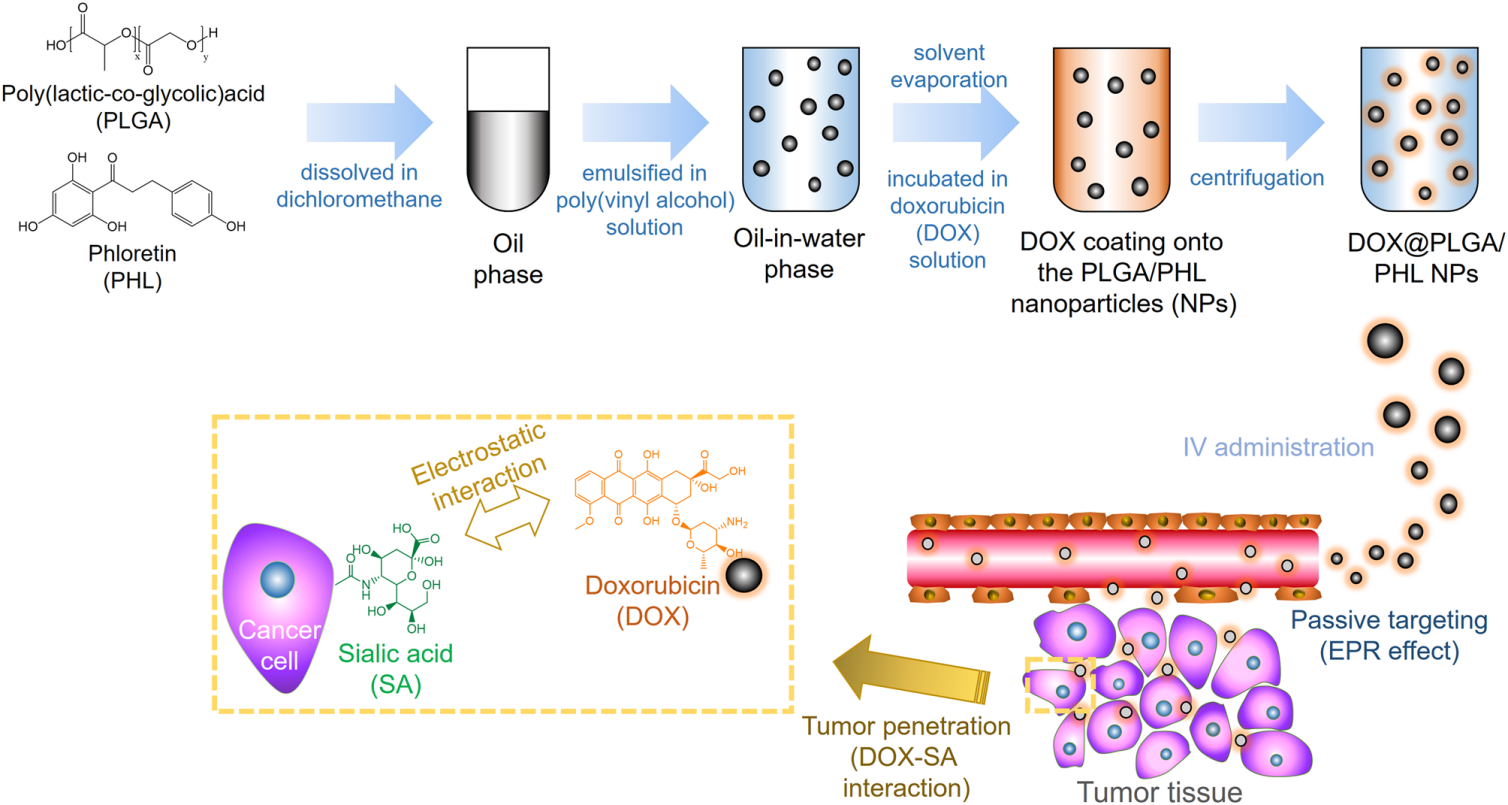
Schematic illustration of SA-assisted tumor targeting strategy of DOX@PLGA/PHL NPs.

PHL was loaded to PLGA NPs as an anticancer agent and DOX was coated onto the outer layer of PLGA/PHL NPs aiming at elevated cellular accumulation, tumor infiltration, and antiproliferation in this study (Fig. 1). Both poorly water-soluble PLGA and PHL can form nano-sized particles via an emulsification-solvent evaporation method^41^. Around the neutral pH range, DOX was conveniently adsorbed onto the outer layer of PLGA/PHL NPs via an electrostatic interaction between positive charge of DOX and negative charge of PLGA/PHL NPs. DOX was introduced to the outer surface of PLGA/PHL NPs for improved tumor targeting and penetration probably based on DOX-SA interactions in this study.

**Figure 2:**
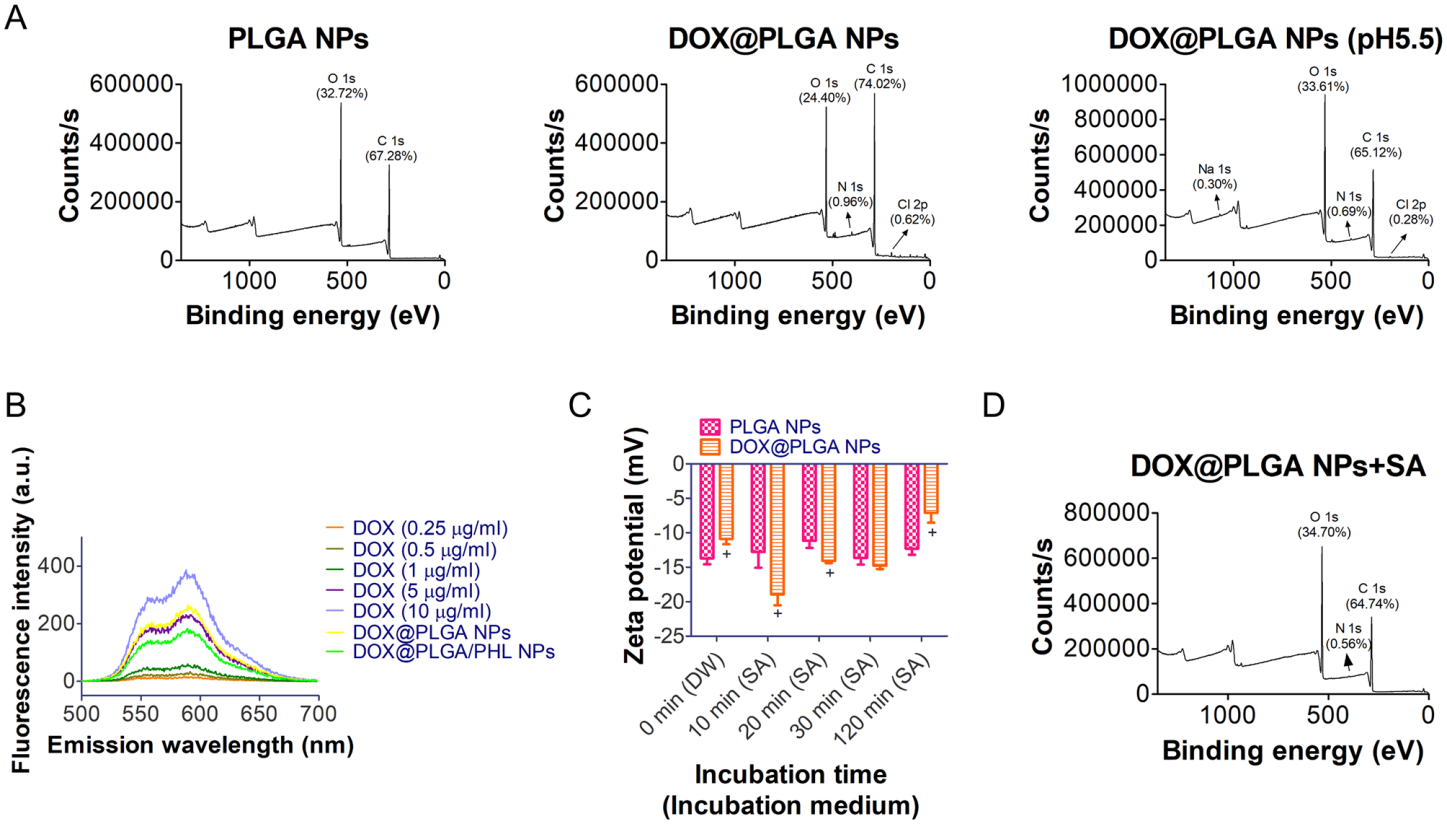
Identification of DOX coating onto PLGA NPs and their interactions with SA. (**A**) XPS data of PLGA NPs, DOX@PLGA NPs, and DOX@PLGA NPs (after incubation at pH 5.5). Binding energy-dependent counts/s values are shown. Atomic contents are presented in the graph. (**B**) Fluorescence intensity profiles of DOX (0.25‒10 μg/ml), DOX@PLGA NPs, and DOX@PLGA/PHL NPs. Emission spectra (500‒700 nm) of all samples at 480 nm excitation wavelength are shown. (**C**) Incubation time-dependent zeta potential values of PLGA NPs and DOX@PLGA NPs after mixing with SA. Zeta potential (mV) values at each determined time (0, 10, 20, 30, and 120 min) are shown. Each point indicates the mean ± SD (*n* = 3). ^+^*p* < 0.05, compared with PLGA NPs group. (**D**) XPS data of DOX@PLGA NPs after incubating with SA. Atomic contents are present in the graph.

The existence of DOX in the outer layer of PLGA NPs was verified by X-ray photoelectron spectroscopy (XPS) analysis and fluorescence measurement (Fig. 2A and 2B). The existence of DOX in the exterior part of developed NP structure was further investigated with XPS analysis (Fig. 2A). XPS data of NP samples can reveal the types and contents of atoms located in the outer layers^42–46^. In case of PLGA, it is composed of only C, H, and O atoms thus peaks for C 1 s and O 1 s were shown in the profile of PLGA NPs group. On the contrary, DOX@PLGA NPs group exhibited the existence of N 1 s and Cl 2p which may be originated from adsorbed DOX HCl molecules. Together with showing up of N 1 s and Cl 2p, the alteration in contents of C 1 s and O 1 s indicates the location of DOX molecules on the outer surface of NPs. Following incubation at pH 5.5 which implying the internal pH of cancer cells, the atomic percentage of N 1 s was attenuated and those of C 1 s and O 1 s were also altered. Attached DOX HCl molecules seem to be liberated at pH 5.5 (due to its higher solubility at acidic pH rather than neutral pH) and it affects the atomic composition of designed DOX@PLGA NPs. It may further support the DOX release in the cancer cells following entry of fabricated DOX@PLGA NPs.

By using the fluorescence property of DOX, the contents of DOX in DOX@PLGA NPs and PHL-loaded DOX@PLGA NPs (DOX@PLGA/PHL NPs) were quantitatively determined (Fig. 2B). Emission spectra (at fixed excitation wavelength) of DOX standard samples, DOX@PLGA NPs, and DOX@PLGA/PHL NPs were acquired and fluorescence intensity at 589.5 nm emission wavelength was used for the calculation of DOX contents in developed NPs. The contents of DOX in DOX@PLGA NPs and DOX@PLGA/PHL NPs were 6.8% and 4.6%, respectively. Electrostatic interactions between cationic DOX and anionic PLGA NPs can be one of major drug adsorption mechanisms onto the outer layer of NP structures. Both XPS and fluorescence spectroscopy data imply the successful introduction of DOX onto the PLGA NPs in this study.

Interactions between DOX (in DOX@PLGA NPs) and SA (as a free molecule) were verified by zeta potential measurement and XPS analyses (Fig. 2C and 2D). Before mixing with SA solution, mean zeta potential values of PLGA NPs and DOX@PLGA NPs were − 13.7 mV and − 10.9 mV, respectively (Fig. 2C). DOX coating onto PLGA NPs rendered less negative charge due to the cationic property of DOX (*p* < 0.05). After blending with SA solution, PLGA NPs group exhibited no significant difference in zeta potential values irrespective of the incubation time. However, the zeta potential value of DOX@PLGA NPs at 10 min (− 18.9 mV) was significantly far from the neutral value rather than that of 0 min group (− 10.9 mV) (*p* < 0.05). It implies the adsorption of SA onto the DOX@PLGA NPs probably based on electrostatic interactions between DOX and SA. At longer incubation time, the zeta potential value of DOX@PLGA NPs group moved to neutral charge, indicating the dissociation of SA sheath from DOX@PLGA NPs. PLGA can be degraded by the hydrolysis of its ester linkage in the aqueous environment and it can be lead to the degradation of NPs. Therefore, SA sheath can be detached from the surface of NPs and the zeta potential value shifted to the neutral charge in longer incubation time. The coating of SA onto the DOX@PLGA NPs was also investigated by XPS analysis (Fig. 2D). Compared to XPS data of DOX@PLGA NPs group (Fig. 2B), the atomic contents of C 1s, O 1s, and N 1s were changed and that of Cl 2p was not detected in (DOX@PLGA NPs + SA) group. Considering the chemical structure of SA, it also supports the existence of SA in the outer layer of (DOX@PLGA NPs + SA) group.

**Figure 3:**
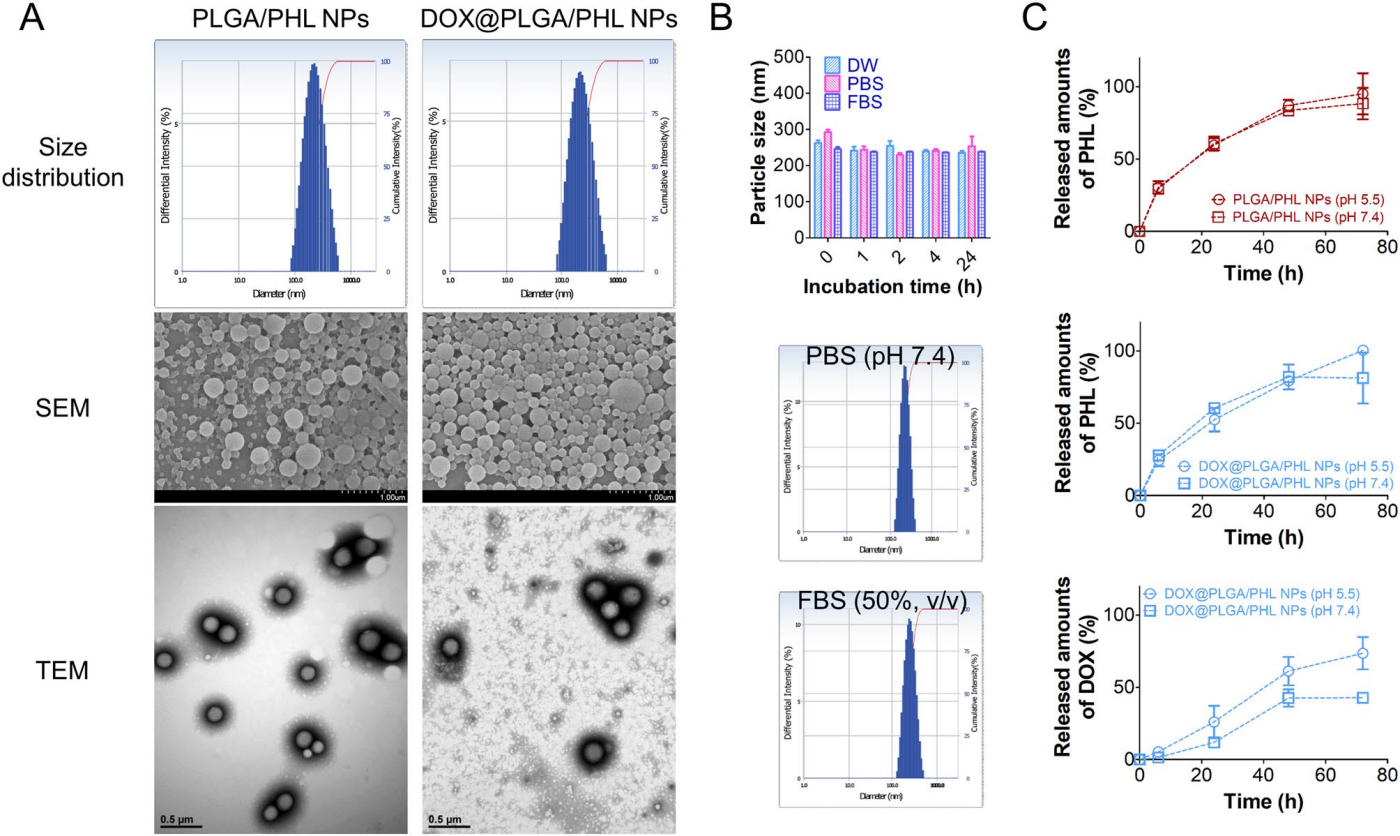
Particle properties of developed NPs. (**A**) Size distribution profiles, SEM images, and TEM images of PLGA/PHL NPs and DOX@PLGA/PHL NPs. Diameter-dependent differential intensity (%) profiles are shown as size distribution diagrams. (**B**) Incubation time-dependent particle size values of DOX@PLGA/PHL NPs in different media (DW, PBS (pH 7.4), and FBS (50%, v/v)). Each point indicates the mean ± SD (*n* = 3). Particle size distribution profiles, shown as diameter-dependent differential intensity values, of DOX@PLGA/PHL NPs in PBS (pH 7.4) and FBS (50%, v/v) mixture are shown. (**C**) Release profiles of PHL and DOX from PLGA/PHL NPs and DOX@PLGA/PHL NPs at pH 7.4 and 5.5. Each point indicates the mean ± SD (*n* = 3).

Aiming at passive tumor targeting (principally related to EPR effect), particles were designed to have nano-size range in this study. By introducing conventional emulsification-solvent evaporation method^3,41^, PLGA-based NPs containing hydrophobic drug (PHL in this study) were successfully fabricated. Hydrodynamic size and polydispersity index values of DOX@PLGA/PHL NPs were 221 nm and 0.18, respectively (Table 1). In DOX@PLGA/PHL NPs group, the zeta potential value was manifestly moved to neutral value compared to PLGA/PHL NPs group mainly due to the adsorption of cationic DOX molecules onto the outer layer of NPs (*p* < 0.05). As shown in Fig. 3A, unimodal size distribution pattern was observed in both PLGA/PHL NPs and DOX@PLGA/PHL NPs groups. Scanning electron microscope (SEM) and transmission electron microscopy (TEM) images also indicate the spherical shape and corresponding similar mean diameter (observed by dynamic light scattering (DLS) method) of both NPs groups (Fig. 3A). The maintenance of constant particle size of DOX@PLGA/PHL NPs in different media was investigated (Fig. 3B). The initial hydrodynamic size of DOX@PLGA/PHL NPs was maintained even after incubating in distilled water (DW), phosphate-buffered saline (PBS, pH 7.4), or fetal bovine serum (FBS) for 24 h. Unimodal particle size distribution pattern was also shown in PBS (pH 7.4) and FBS (50%, v/v) groups at 24 h. Constant particle size of DOX@PLGA/PHL NPs in PBS (pH 7.4) and FBS (50%, v/v) may imply the absence of aggregation or agglomeration of NPs in the biological fluids (*i.e.,* blood) after intravenous injection. It may guarantee the efficient passive tumor targeting strategy which is mainly governed by the physicochemical properties of particles and the anatomical structures of cancer tissues.

**Table 1:** Particle characterization of NPs.

Formulation	Mean diameter (nm)	Polydispersity index	Zeta potential (mV)	PHL encapsulation efficiency (%)^a^
PLGA/PHL NPs	211 ± 19	0.14 ± 0.04	− 15.9 ± 2.0	56.5 ± 0.4
DOX@PLGA/PHL NPs	221 ± 11	0.18 ± 0.02	− 8.4 ± 0.7*	42.4 ± 0.3

*p < 0.05, compared with PLGA/PHL NPs.

Data are shown as mean ± standard deviation (SD) (*n* = 3).

NPs were dispersed in DW at 5 mg/ml.

^a^ $${\text{Encapsulation}}\;{\text{efficiency}}\,(\% ) = \frac{{{\text{Actual}}\,{\text{amount}}\,{\text{of}}\,{\text{drug}}\,{\text{in}}\,{\text{NPs}}}}{{{\text{Input}}\,{\text{amount}}\,{\text{of}}\,{\text{drug}}\,{\text{in}}\,{\text{NPs}}}} \times 100.$$

PHL and DOX release patterns from designed NPs were tested at pH 7.4 and 5.5 (Fig. 3C), indicating the normal physiological conditions and intracellular environment of cancer cells. Sustained drug release patterns were observed during the testing period in both formulation groups. DOX coating onto the surface of PLGA/PHL NPs did not evidently affect the release pattern of PHL in this study. In addition, pH value (pH 7.4 vs 5.5) of release media did not significantly influence on the release profiles of PHL from both types of NPs. On the contrary, DOX release from DOX@PLGA/PHL NPs was elevated at pH 5.5 rather than pH 7.4, probably due to its higher solubility in acidic pH^21^. It indicates the selective detachment of DOX from the outer surface of NPs and its consecutive release inside the cancer cells (pH 5.5) rather than the other normal organs and tissues (pH 7.4). The release rate of adsorbed DOX was relatively slow and it might be due to its another salt formation in PBS (used as a release medium in this study). Together with this, slow diffusion of DOX across the dialysis membrane as estimated from the diffusion data of free DOX (Fig. S1) also might contribute to the sustained release of DOX from designed DOX@PLGA/PHL NPs^47^. Observed sustained drug release property may attribute to the reduction of dosing frequency and improvement of patient compliance.

### Cellular internalization and localization

**Figure 4:**
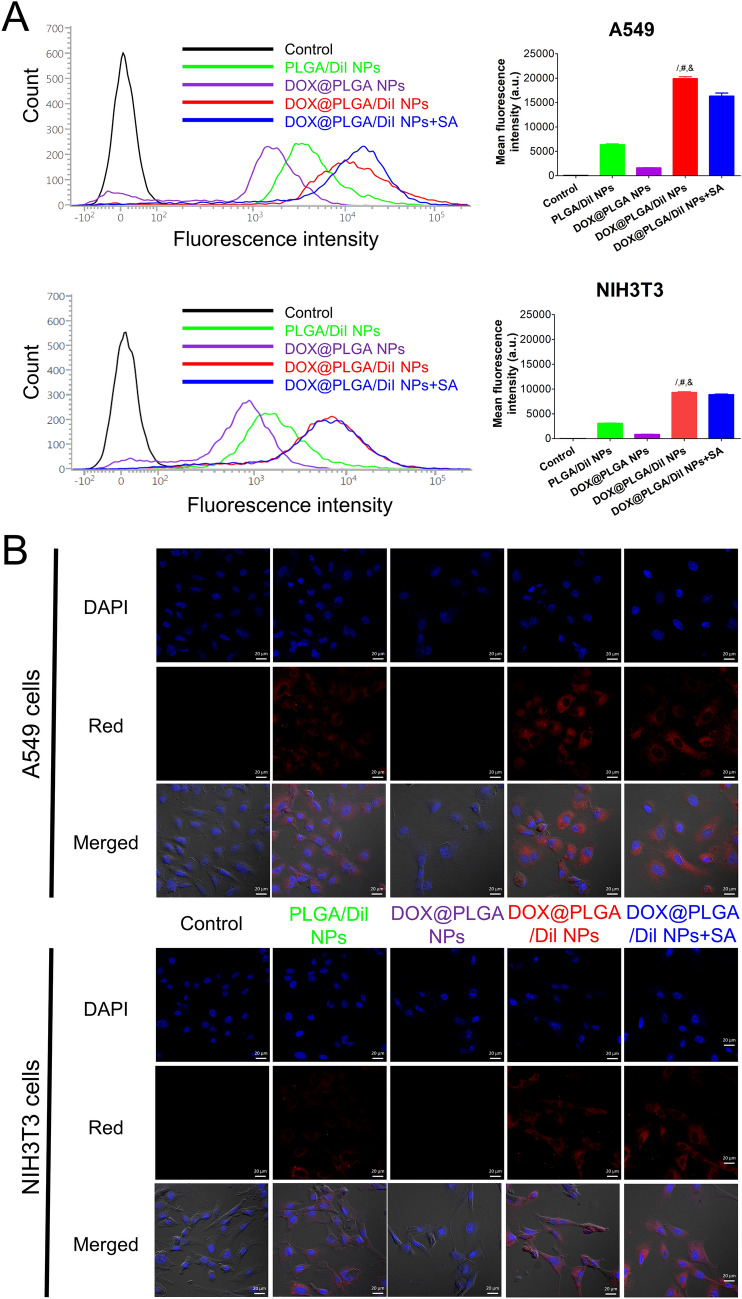
Cellular accumulation and distribution patterns of developed NPs in A549 and NIH3T3 cells. (**A**) Cellular uptake data of Dil-loaded NPs in A549 and NIH3T3 cells analyzed by flow cytometry. Cell count values according to the fluorescence intensity of control, PLGA/Dil NPs, DOX@PLGA NPs, DOX@PLGA/Dil NPs, and DOX@PLGA/Dil NPs + SA groups are shown. Mean fluorescence intensity values of all experimental groups are plotted. Each point indicates the mean ± SD (*n* = 3). ^/^*p* < 0.05, compared with PLGA/Dil NPs group. ^#^*p* < 0.05, compared with DOX@PLGA NPs group. ^&^*p* < 0.05, compared with DOX@PLGA/Dil + SA NPs group. (**B**) Intracellular fluorescence signals of developed NPs in A549 and NIH3T3 cells observed by CLSM imaging. DAPI, red, and merged images of control, PLGA/Dil NPs, DOX@PLGA NPs, DOX@PLGA/Dil NPs, and DOX@PLGA/Dil NPs + SA groups are shown. The length of scale bar in the image is 20 μm.

Enhanced cellular accumulation of PLGA NPs by DOX coating and the feasible contribution of SA, which is overexpressed in cancer cells rather than healthy cells, was assessed by flow cytometry and confocal laser scanning microscopy (CLSM) imaging analyses (Fig. 4). For fluorescence detection of designed NP structures, 1,1′-dioctadecyl-3,3,3′,3′-tetramethylindocarbocyanine perchlorate (Dil) was encapsulated in PLGA NPs as a fluorescence dye. Dil was successfully entrapped in PLGA NPs and the intracellular movement of NPs can be estimated from the fluorescence signals of Dil as reported^41,48–51^. In current testing conditions, Dil group exhibited much higher fluorescence intensity compared to DOX group in both A549 and NIH3T3 cells (Fig. S2). As shown in Fig. 4A, the mean fluorescence intensity value of DOX@PLGA/Dil NPs group was 3.1-fold higher than PLGA/Dil NPs group (*p* < 0.05). Considering much lower cellular fluorescence intensity of DOX rather than Dil (Fig. S2), DOX adsorbed onto the outer layer of PLGA/Dil NPs seems to obviously improve the endocytosis efficiency of PLGA/Dil NPs in A549 cells, irrespective of the intrinsic fluorescence signal of DOX. The mean fluorescence intensity of DOX@PLGA NPs group was only 8.1% of that in DOX@PLGA/Dil NPs group. It means that most of fluorescence signal was derived from Dil encapsulated in DOX@PLGA/Dil NPs, rather than DOX located in the outer surface of DOX@PLGA/Dil NPs. For demonstrating DOX-related cellular uptake mechanisms, SA was selected and its contribution to the cellular entry process was assessed with inhibition study. SA and DOX@PLGA/Dil NPs were pre-mixed prior to their treatment to the cells for accumulation tests. Added SA may be bound to the outer surface of DOX@PLGA/Dil NPs and that may hamper cellular SA-mediated endocytosis of DOX@PLGA/Dil NPs. Therefore, it is expected that the internalization efficiency of SA-adsorbed DOX@PLGA/Dil NPs may be lower than DOX@PLGA/Dil NPs. Actually, pre-incubation with SA induced 18.0% reduction of the mean fluorescence intensity compared to DOX@PLGA/Dil NPs group (*p* < 0.05). It implies that SA located in A549 cells may interact with DOX existed in DOX@PLGA/Dil NPs, resulting in an improved cellular accumulation in cancer cells. As the pK_a_ value of carboxylic acid in SA is 2.6, it can have a negative charge in physiological pH range^40^. The pK_a_ value of primary amine group in DOX is reported to be 8.2–9.9^33–35^, thus it may show a positive charge at acidic and neutral pH indicating the condition of intracellular compartment, cancer cells, and normal cells, respectively. Therefore, DOX-SA complex might be formed based on the electrostatic interactions at acidic and neutral pH range (from 5.5 to 7.4) and it can contribute to the elevated cellular entry of DOX@PLGA NPs in this study. NIH3T3 cells were selected as fibroblast cells for verifying SA-assisted enhanced cellular internalization of DOX-coated NPs. NIH3T3 cells are healthy cells without malignant transformation thus they might have less SA levels on proteins and glycolipids rather than cancer cells. DOX@PLGA/Dil NPs group exhibited 2.1-fold higher fluorescence intensity in A549 cells rather than NIH3T3 cells. In addition, the pre-incubation of DOX@PLGA/Dil NPs with SA reduced only 4.7% reduction in fluorescence intensity compared to that of DOX@PLGA/Dil NPs group in NIH3T3 cells. Considering higher expression level of SA in malignant cells (A549 cells) rather than non-cancer cells (NIH3T3 cells)^38,39^, designed DOX-engineered PLGA NPs seem to be efficiently internalized into the cancer cells with the assistance of SA. Cellular distribution of Dil-loaded NP structures was also monitored by CLSM imaging (Fig. 4B). Red cellular fluorescence signals in DOX@PLGA/Dil NPs were much stronger than those of PLGA/Dil NPs and DOX@PLGA NPs. Similar cellular accumulation patterns as observed in flow cytometer analysis were present in NIH3T3 cells. These cellular accumulation and distribution data suggest the feasible attribution of SA to the cellular entry of designed DOX@PLGA NPs.

### *In vitro *antiproliferation activities

**Figure 5:**
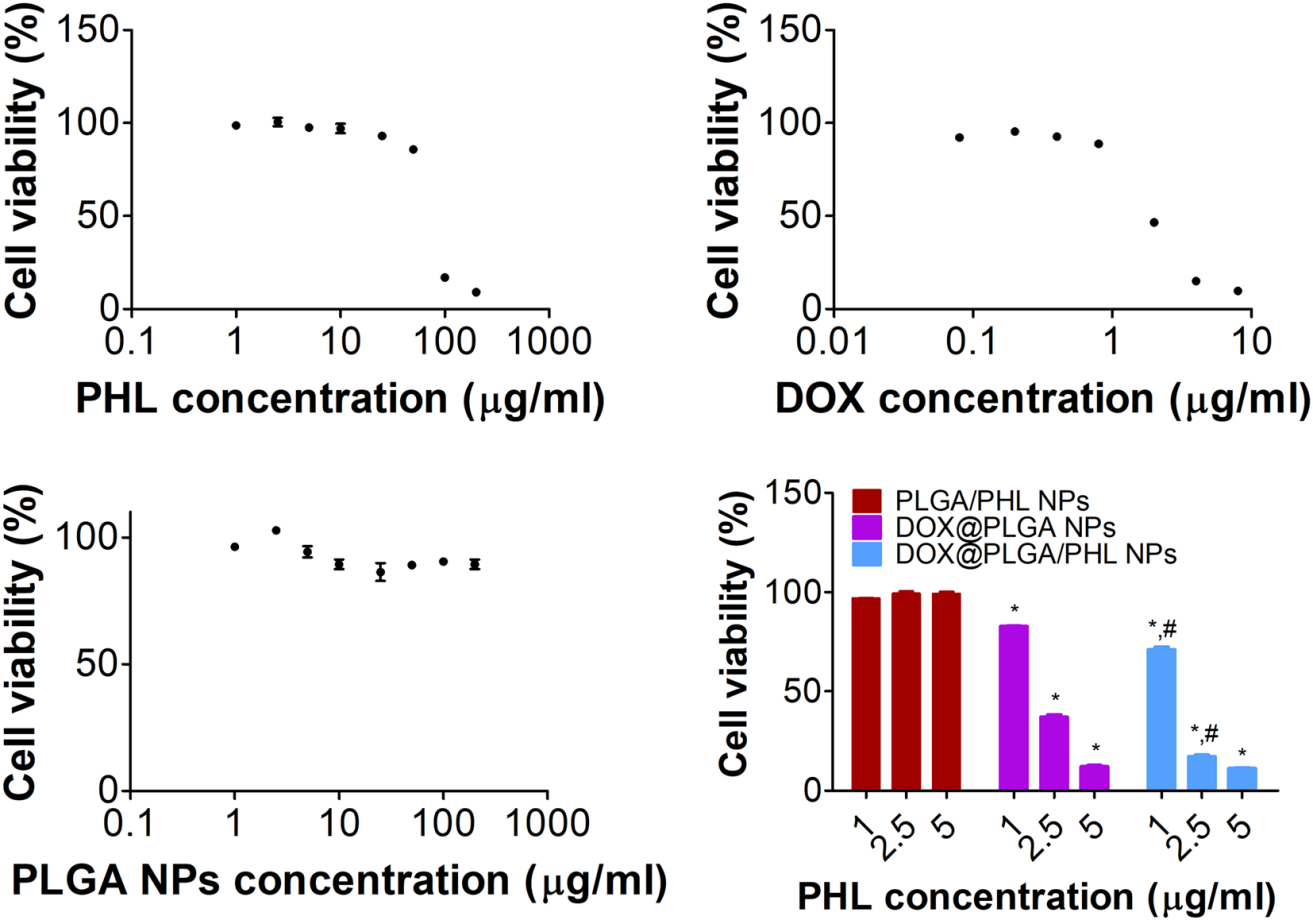
Cytotoxicity tests in A549 cells. Cell viability profiles according to PHL, DOX, and PLGA NPs concentrations are shown. Antiproliferation potentials of PLGA/PHL NPs, DOX@PLGA NPs, and DOX@PLGA/PHL NPs at 1, 2.5, and 5 μg/ml PHL concentrations are exhibited. Each point indicates the mean ± SD (*n* = 3). ^*^p < 0.05, compared with PLGA/PHL NPs group. ^#^*p* < 0.05, compared with DOX@PLGA NPs group.

Cytotoxicity profiles of NP structures and their components were acquired by colorimetric assay in A549 cells (Fig. 5). DOX and PHL included in developed NPs can act as anticancer agents against A549 cells in this study. Mean IC_50_ values of PHL and DOX in A549 cells were 97.3 and 3.3 μg/ml, respectively, in current experimental conditions. Together with clinically approved DOX, PHL exhibited antiproliferation potentials in various types of cancer cells mainly based on the inhibition of glucose transporters^41,52–54^. In case of PLGA NPs, they did not show severe cytotoxicity (over 90% cell viability) in tested concentration range (up to 200 μg/ml). Antiproliferation potentials of PLGA/PHL NPs, DOX@PLGA NPs, and DOX@PLGA/PHL NPs groups were assessed at 1‒5 μg/ml PHL concentration range in A549 cells. DOX@PLGA/PHL NPs group exhibited apparently lower cell viability values rather than DOX@PLGA NPs at 1 and 2.5 μg/ml concentrations (*p* < 0.05). It may be due to the combinatorial effects of PHL and DOX. Of note, DOX located in the outer surface of NPs seems to play more critical roles, rather than encapsulated PHL, in killing cancer cells. It is speculated that designed DOX@PLGA/PHL NPs can be efficiently applied to inhibit the proliferation of A549 cells.

### Tumor penetration potentials of NPs in spheroid models

**Figure 6:**
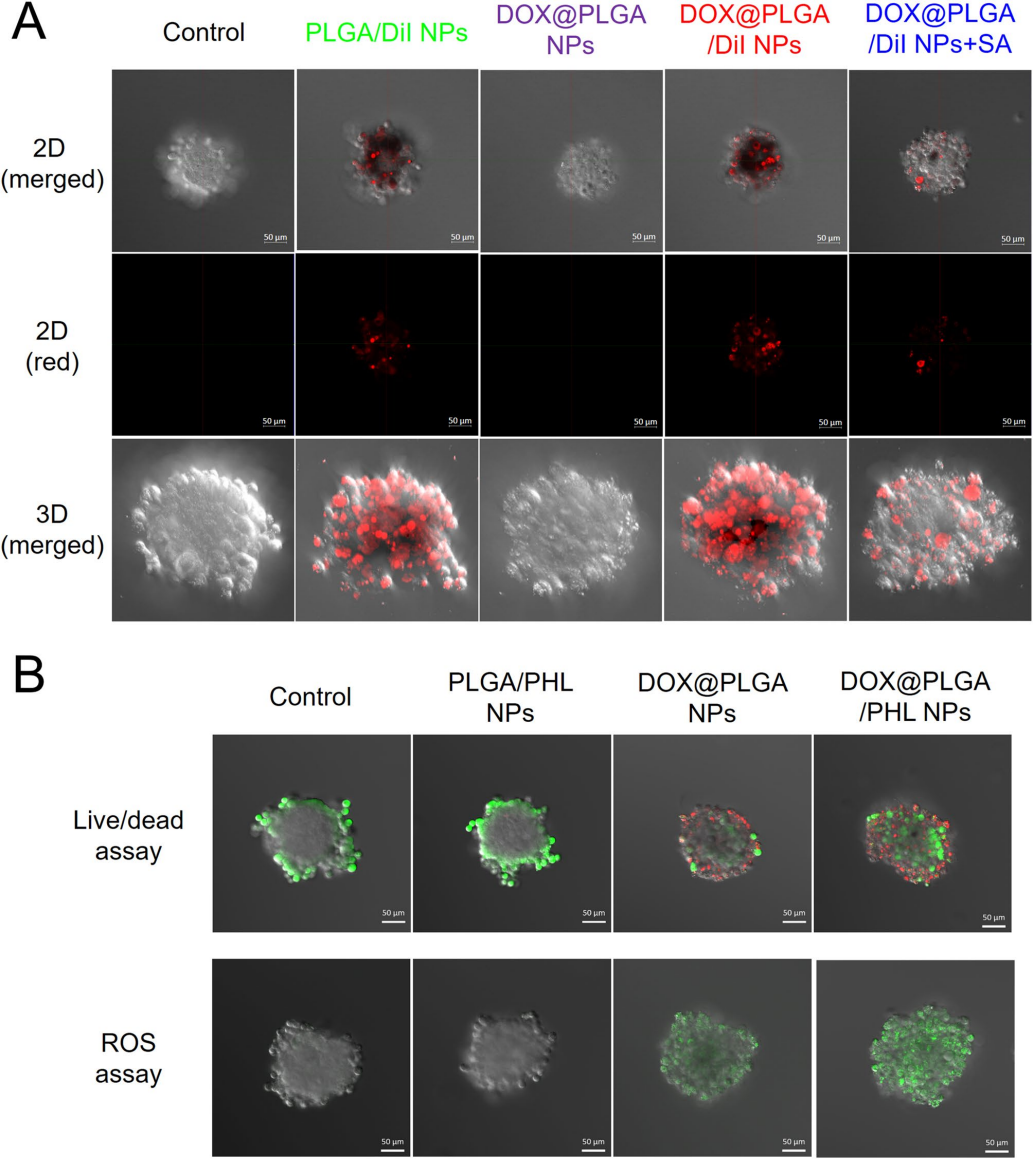
Tumor penetration capability and anticancer activity tests of developed NPs in A549 multicell spheroid model. (**A**) Tumor infiltration efficacy test of Dil-loaded NPs in A549 spheroid model. 2D (merged), 2D (red), and 3D (merged) images of control, PLGA/Dil NP, DOX@PLGA NPs, DOX@PLGA/Dil NPs, and DOX@PLGA/Dil NPs + SA groups are shown. The length of scale bar in the image is 50 μm. (**B**) Spheroid images after applying live/dead assay and ROS assay reagents observed by CLSM imaging. Merged images of control, PLGA/PHL NPs, DOX@PLGA NPs, and DOX@PLGA/PHL NPs are shown. The length of scale bar in the image is 50 μm.

Tumor penetration capability of designed NP structures was assessed in A549 cell spheroid model^21^. SA has been abundantly expressed in tumor tissues rather than normal tissues^38,39^, therefore SA can be used as one of targets for the development of NPs aiming at improved tumor infiltration^21,37,55^. As shown in Fig. 6A, PLGA/Dil NPs group exhibited red fluorescence signals in the exterior boundary of A549 tumor spheroid. Although its intensity was much stronger than that of DOX@PLGA NPs group, it was weaker than that of DOX@PLGA/Dil NPs group. DOX@PLGA/Dil NPs group have the most efficient tumor penetration potential according to the fluorescence levels in the spheroid model. In 2D and 3D images, the fluorescence intensity values of (DOX@PLGA/Dil NPs + SA) group were 42% and 29% of DOX@PLGA/Dil NPs group, respectively. It means that SA can transport DOX-coated PLGA NPs from outer boundary to core region of A549 tumor spheroids. The observed findings suggest that DOX-SA interaction can be adopted as one of promising tumor penetration strategies. Moreover, SA can provide dual roles of tumor targeting and penetrating potentials in cancer therapy. It is surmised that DOX@PLGA NPs may be efficiently infiltrated into the heterogeneous tumor tissues after their arrival in tumor region following intravenous administration.

Anticancer activities of designed NPs were tested in A549 spheroids by live/dead and ROS assays (Fig. 6B). In CLSM images of live/dead assay, green and red colors indicate live and dead portions, respectively. Portion of dead cells (red color) in DOX@PLGA/PHL NPs was clearly higher than that of PLGA/PHL NPs and DOX@PLGA NPs groups. DOX-SA interactions can guide the efficient infiltration of DOX@PLGA/PHL NPs and elevate their anticancer activities in A549 spheroids. In ROS assay (Fig. 6B), the green fluorescence signal indicates the cellular reactive oxygen species (ROS) level. Notably, the relative ratio of corrected total cell fluorescence of DOX@PLGA/PHL NPs to DOX@PLGA NPs was 2.6 in this study. It implies higher ROS generation capability of DOX@PLGA/PHL NPs compared to the other formulations. It can lead to more efficient cancer cell killing properties in A549 spheroids.

### *In vivo *toxicity

**Figure 7:**
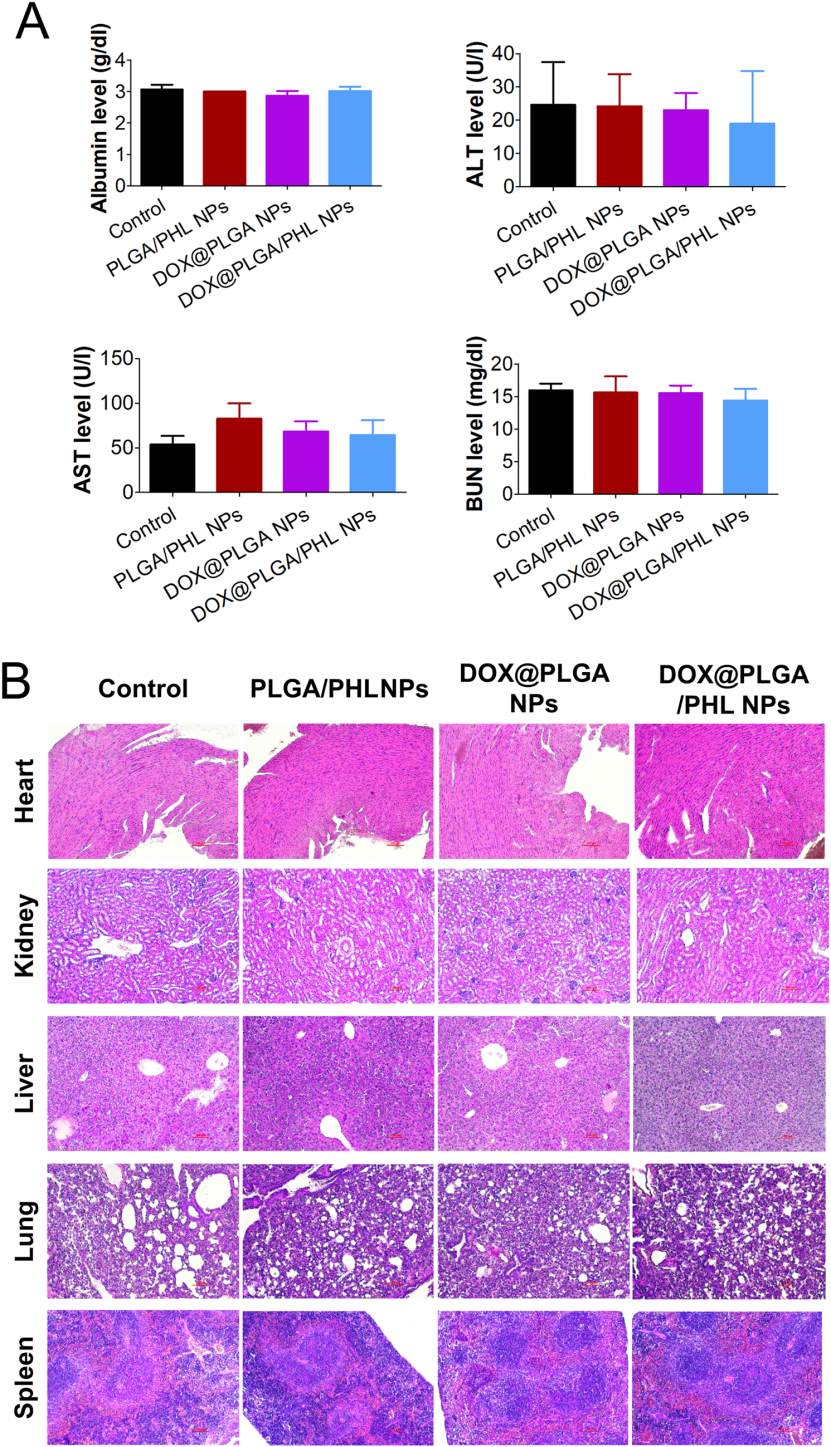
In vivo toxicity data of developed NPs in mouse. (**A**) Serum albumin, ALT, AST, and BUN levels in mouse after intravenous injection. Each point indicates the mean ± SD (*n* = 5). (**B**) H&E staining images of heart, kidney, liver, lung, and spleen in control, PLGA/PHL NPs, DOX@PLGA NPs, and DOX@PLGA/PHL NPs. The length of scale bar in the image is 100 μm.

Systemic toxicity of developed NPs was tested in mice after intravenous injection (Fig. 7). There are some concerns that cationic charged nanocarriers could induce systemic toxicities following intravascular injection^56^. Cationic chemotherapeutic agent (*i.e.,* DOX) was installed in the outer surface of PLGA/PHL NPs in this study. Systemic toxicities of developed NP formulations were evaluated by blood chemistry test and histological staining assay of major tissues and organs. According to the blood chemistry data (Fig. 7A), DOX@PLGA/PHL NPs group did not show any significant differences compared to control group in all markers. Malfunctions in liver and kidney can be estimated from serum albumin/alanine transaminase (ALT)/aspartate transaminase (AST) and blood urea nitrogen (BUN) levels, respectively. No significant difference between control and DOX@PLGA/PHL NPs groups implies the absence of severe toxicities in liver and kidney. In addition, PLGA/PHL NPs and DOX@PLGA NPs also did not induce any dramatic alteration in serum marker levels. It was reported that DOX can induce cardiotoxicity after its injection^57^. However, DOX@PLGA NPs and DOX@PLGA/PHL NPs group did not show obvious histological changes in heart tissues. It can be concluded that DOX included in DOX@PLGA/PHL NPs may not induce fatal cardiotoxicities in current administration dose. As revealed by the blood assay data, any evident morphological changes were not detected in liver, spleen, and kidney specimens (Fig. 7B). Also in lung tissue, DOX@PLGA/PHL NPs group did not show any pathophysiological changes compared to the other experimental groups. All these data support the absence of severe systemic toxicities after multiple dosing in mouse. It can elevate the feasibility for clinical translation of designed NP formulations.

### Tumor targeting capability

**Figure 8:**
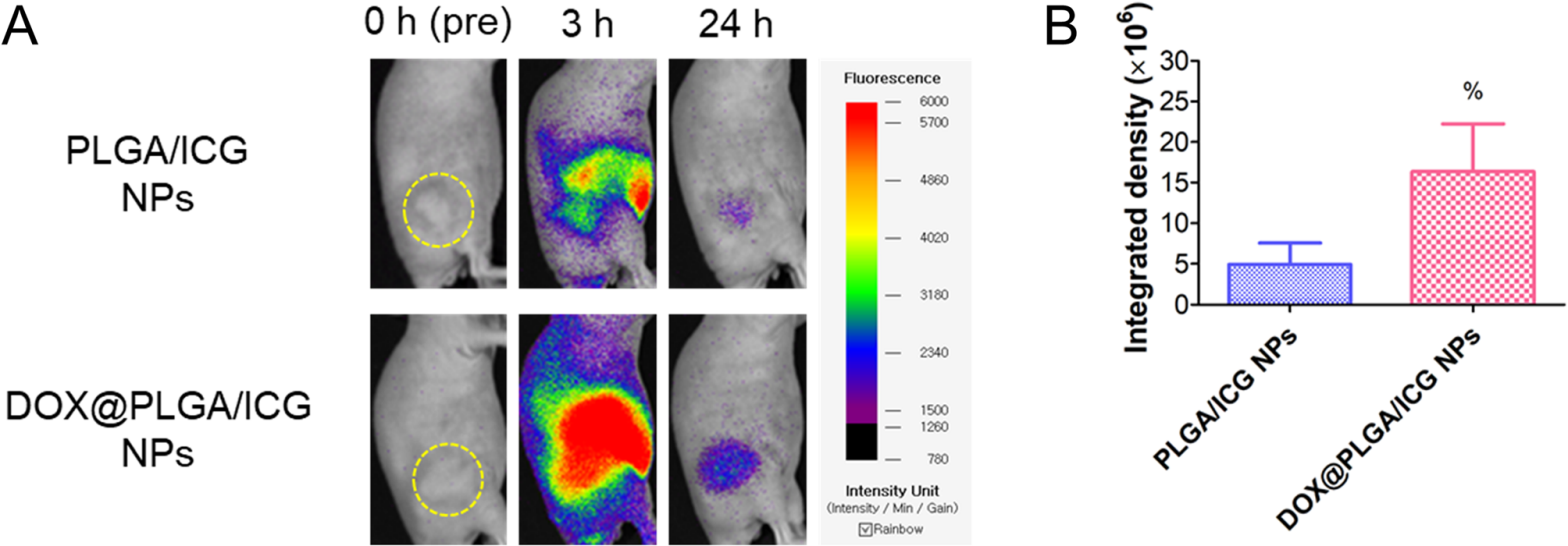
In vivo tumor targeting efficiency of designed NPs. (**A**) Whole body scanned NIRF image of PLGA/ICG NPs and DOX@PLGA/ICG NPs. Yellow dotted circle indicates the tumor region in A549 tumor-bearing mouse model. NIRF images at 0 h (pre-injection), 3 h (post-injection), and 24 h (post-injection) are shown. (**B**) Integrated density of NIRF signals in PLGA/ICG NPs and DOX@PLGA/ICG NPs groups. Each point indicates the mean ± SD (*n* = 3–6). ^%^*p* < 0.05, compared with PLGA/ICG NPs group.

The tumor targeting potentials of DOX-SA interactions were explored in A549 tumor-bearing mouse models by near-infrared fluorescence (NIRF) imaging (Fig. 8). Indocyanine green (ICG) was encapsulated in PLGA NPs as NIRF dye and the in vivo fate of DOX@PLGA/ICG NPs was tracked by NIRF imaging. PLGA/ICG NPs or DOX@PLGA/ICG NPs were injected intravenously to A549 tumor-implanted mouse and whole body NIRF signal was scanned for 24 h (Fig. 8). Integrated density (= area × mean of intensity) of DOX@PLGA/ICG NPs-injected group was 3.3-fold higher than that of PLGA/ICG NPs at 24 h (*p* < 0.05). As demonstrated in A549 cells and spheroids (Figs. 4–6), higher expression of SA in tumor tissue seems to act as a potential target for DOX-coated PLGA NPs^38,39^. Although DOX existed on the surface of DOX@PLGA NPs could bind to other biological components during blood circulation, tumor accumulation of DOX@PLGA NPs was significantly elevated compared to unmodified PLGA NPs. It is expected that designed DOX-engineered PLGA NPs might produce improved anticancer activities following intravenous injection.

## Conclusions

DOX-engineered PLGA NPs containing PHL were developed for SA-assisted tumor targeting and penetration in lung cancer therapy. DOX-adsorbed PLGA/PHL NPs with 221 nm hydrodynamic size, spherical shape, unimodal size distribution, and negative surface charge were fabricated. DOX molecules were attached to PLGA-based NPs via electrostatic interactions and their possible interaction with SA in tumor tissues were adequately demonstrated by XPS and zeta potential analyses. Although there may be other uptake mechanisms of designed DOX@PLGA NPs in cancer cells, the possible contribution of SA expressed in cancer cells was tested in this study. Compared to plain PLGA NPs, DOX@PLGA NPs exhibited improved cellular accumulation and antiproliferation activities. In addition, DOX@PLGA NPs group displayed superior tumor infiltration potentials in A549 tumor spheroid models and better tumor targeting efficiency in A549 tumor bearing mouse models following intravenous injection compared to unmodified PLGA NPs. Considering the negligible toxicity of developed DOX@PLGA/PHL NPs, it can be one of passive and active tumor targeting nanosystems with high feasibility for clinical application.

## Materials and methods

### Materials

DOX was supplied by Boryung Pharmaceutical Co., Ltd. (Seoul, Korea). PLGA (M_n_: 45‒55 kDa; LA:GA = 50:50) was obtained from PolySciTech (Akina, Inc., West Lafayette, IN, USA). ICG, PHL, and sodium dodecyl sulfate (SDS) were acquired from Tokyo Chemical Industry Co. Ltd. (Tokyo, Japan). Dil, poly(vinyl alcohol) (PVA; 30–70 kDa molecular weight (MW)), and SA (*N*-acetylneuraminic acid) were supplied by Sigma–Aldrich (St. Louis, MO, USA). Dulbecco’s modified Eagle’s medium (DMEM), RPMI 1640 (developed by Roswell Park Memorial Institute), penicillin–streptomycin, and FBS were provided by Life Technologies, Corp. (Carlsbad, CA, USA). All other reagents were analytical grade.

### Preparation of NPs

NPs mainly composed of PLGA were fabricated by an emulsification-evaporation method as reported^3,29,41,48,51,58,61^. PLGA (40 mg) dissolved in dichloromethane (1.6 ml) was mixed with dimethyl sulfoxide (DMSO, 0.4 ml). It was added to 0.5% (w/v) PVA solution (20 ml) and that emulsion was homogeneously mixed with an ultrasonic liquid processor (VC-750; Sonics & Materials, Inc., Newtown, CT, USA) for 10 min. That was stirred for 2 h at room temperature for eliminating organic solvents. The pellet of NPs was obtained by centrifugation at 16,100 g for 30 min. For preparing PLGA NPs, half part of resuspension of NP pellet was lyophilized. To prepare DOX@PLGA NPs, other half part of NP pellet was resuspended with DOX solution (20 mg) in DW (1 ml) and incubated for 1 h. Then, it was centrifuged at 16,100 g for 30 min and the resuspension of NP pellet was freeze-dried.

PLGA (40 mg) in dichloromethane (1.6 ml) and PHL (8 mg) in DMSO (0.4 ml) were blended and it was added to 0.5% (w/v) PVA solution (20 ml). It was homogeneously mixed with an ultrasonic liquid processor (VC-750; Sonics & Materials, Inc., Newtown, CT, USA) for 10 min. That emulsion was stirred for 2 h at room temperature to remove the organic solvents. NP pellet was acquired by centrifugation at 16,100 g for 30 min. Half of NP suspension was lyophilized to make dried PLGA/PHL NPs. The other half part of NP suspension was incubated in DOX (10 mg) in DW (0.5 ml) for 1 h to prepare DOX@PLGA/PHL NPs. NP pellet was obtained by centrifugation at 16,100 g for 30 min and it was freeze-dried for further uses.

### Identification assays of DOX coating onto the NPs and interactions between DOX and SA

The atomic percentages in the outer layers of PLGA NPs, DOX@PLGA NPs, and DOX@PLGA NPs (after incubating at PBS (pH 5.5) for 3 days) were determined by XPS (K-Alpha^+^, Thermo Fisher Scientific, Waltham, MA, USA)^60^. The percentages of C (1 s), O (1 s), Cl (2p), N (1 s), and Na (1 s) in PLGA NPs, DOX@PLGA NPs, and DOX@PLGA NPs (after incubating at pH 5.5) were measured.

DOX coating onto NPs was identified by fluorescence intensity measurement. DOX (0.25, 0.5, 1, 5, and 10 μg/ml in DMSO), DOX@PLGA NPs (100 μg/ml in DMSO), and DOX@PLGA/PHL NPs (100 μg/ml in DMSO) samples were prepared. The emission spectra (at 500–700 nm wavelength range) were measured at 480 nm excitation wavelength by using a fluorescence spectrometer (LS-55, PerkinElmer, Inc., Waltham, MA, USA). By using linear regression line based on fluorescence values of DOX standard samples, the contents of DOX in DOX@PLGA NPs and DOX@PLGA/PHL NPs were determined.

Zeta potential values of PLGA NPs and DOX@PLGA NPs after incubation with SA for 120 min were measured by laser Doppler methods (ELS-Z1000; Otsuka Electronics, Tokyo, Japan). Dispersion of freeze dried NPs (10 mg/ml in DW) and SA solution (1 mg/ml in DW) were mixed at equivalent volume and that mixture was centrifuged at 16,100 g for 30 min. Supernatant was discarded and NP pellet was dispersed in DW for measuring zeta potential values.

Binding potential of DOX@PLGA NPs and SA was also evaluated by XPS analysis. DOX@PLGA NPs in DW (20 mg/ml) and SA in DW (2 mg/ml) were mixed at 1:1 volume ratio and incubated for 2 h at room temperature. That mixture was centrifuged at 16,100 g for 30 min and the supernatant was discarded. After repeating that washing process one more time, the pellet was lyophilized. The atomic percentages in DOX@PLGA NPs + SA group were analyzed by aforementioned XPS analysis.

### Particle characterization of NPs

The particle characteristics, such as hydrodynamic size, polydispersity index, and zeta potential, of PLGA/PHL NPs and DOX@PLGA/PHL NPs were determined by using DLS and laser Doppler methods (ELS-Z1000; Otsuka Electronics) in accordance with the manufacturer’s protocol^41,59,60^. The morphological shape of PLGA/PHL NPs and DOX@PLGA/PHL NPs were monitored with ultrahigh resolution scanning electron microscope (UHR-SEM; S-4800, Hitachi, Tokyo, Japan)^60^. Particle shape of PLGA/PHL NPs and DOX@PLGA/PHL NPs was further observed by TEM. NPs were stained with 2% (w/v) phosphotungstic acid, placed on the copper grids with films, destained with DW, and dried for 10 min, prior to the observation by using TEM (JEM 1010; JEOL, Tokyo, Japan)^59^.

Encapsulation efficiency values of PHL in PLGA/PHL NPs and DOX@PLGA/PHL NPs were measured with high performance liquid chromatography (HPLC) system^60^. HPLC system (1260 Infinity II, Agilent Technologies, Santa Clara, CA, USA) composed of autosampler (1260 Vialsampler), pump (1260 Quat Pump VL), and UV/Vis detector (1260 VWD) connected with a reverse phase C18 column (Gemini, 250 mm × 4.6 mm, 5 μm; Phenomenex, Torrance, CA, USA) was used for the analysis of PHL. Mobile phase was prepared with the mixture of acetonitrile, DW, and phosphoric acid (50:50:0.08, v/v/v). The flow rate and injection volume were set at 1 ml/min and 20 μl, respectively. PLGA/PHL NPs or DOX@PLGA/PHL NPs were dissolved in DMSO and further diluted with the mobile phase. The absorbance of eluent was detected at 288 nm.

Particle stability of DOX@PLGA/PHL NPs was tested by measuring the hydrodynamic size^41,62^. NPs dispersed in DW (10 mg/ml) was mixed with DW, PBS (pH 7.4), or FBS at an equivalent volume ratio. Hydrodynamic size and particle size distribution were measured by described DLS method at 0, 1, 2, 4, and 24 h post-incubation.

PHL and DOX release profiles from PLGA/PHL NPs and DOX@PLGA/PHL NPs were tested at pH 7.4 and 5.5^60^. DOX diffusion profiles across the dialysis membrane were also studied at pH 7.4 and 5.5. Dispersion (0.15 ml) of NPs containing 150 μg PHL or solution (0.15 ml) of free DOX (corresponding DOX amount in DOX@PLGA/PHL NPs (150 μg PHL)) was put into Mini-GeBAflex tubes (MWCO: 14 kDa; Gene Bio-Application Ltd., Yavne, Israel). That dialysis tube was transferred to conical tube containing 10 ml PBS (pH 7.4 or 5.5) including 0.3% SDS with 50 rpm agitation at 37 °C. At pre-determined time (6, 24, 48, and 72 h for NPs and 2, 6, 24, and 48 h for free DOX), release media (0.2 ml) were collected and the equivalent volume of fresh media was supplemented. PHL concentrations in the collected media were determined by the described HPLC method. DOX concentrations in the media were analyzed by fluorescence spectroscopy method. Fluorescence signals were detected at 480 nm (excitation) and 560 nm (emission) wavelengths by using a multi-mode microplate reader (SpectraMax i3, Molecular Devices, Sunnyvale, CA, USA).

### Cellular uptake and distribution studies

Cellular accumulation and localization patterns of developed NPs were assessed in A549 cell (human lung adenocarcinoma cell; Korean Cell Line Bank (KCLB), Seoul, Korea) and NIH3T3 cell (mouse embryonic fibroblast cells; KCLB). A549 cells were cultured in RPMI 1640 (containing 300 mg/l of L-glutamine) including heat inactivated FBS (10%, v/v) and penicillin–streptomycin (1%, v/v) and NIH3T3 cells were cultured with DMEM containing 10% (v/v) FBS and 1% (v/v) penicillin–streptomycin, respectively. For fluorescence detection by flow cytometry and CLSM, Dil was loaded to PLGA NPs as a fluorescence dye. For the fabrication of PLGA/Dil NPs and DOX@PLGA/Dil NPs, Dil (1 mg) and PLGA (40 mg) were dissolved in the organic phase and poured into the 0.5% (w/v) PVA solution (20 ml) for making oil-in-water type emulsion. Following procedures were identical with the described method in above section.

A549 or NIH3T3 cells were seeded at a density of 4.0 × 10^5^ cells per well in six-well plates and cultured at 37 °C for 1 day. Dil, DOX, Dil + DOX, PLGA/Dil NPs, DOX@PLGA NPs, and DOX@PLGA/Dil NPs were added to the cells at 1 μg/ml Dil concentration and/or corresponding DOX concentration (1.16 μg/ml) and incubated for 24 h. In case of DOX@PLGA/Dil NPs + SA group, DOX@PLGA/Dil NPs were pre-incubated with SA (150 μg/ml) prior to their application to the cells. Samples were eliminated and the cells were rinsed with PBS (pH 7.4) at least thrice. Cells were detached from the plate and cell pellets were obtained by centrifugation at 1,000 g for 5 min. Those cells were then suspended in FBS (2%, v/v) solution for flow cytometry analysis. Fluorescence intensity-dependent cell count was detected by a FACSCalibur Fluorescence-activated Cell Sorter (FACS) (BD Biosciences, San Jose, CA, USA)^62^.

Cellular distribution of designed NPs was evaluated by CLSM imaging. A549 or NIH3T3 cells were seeded on culture slides (BD Falcon, Bedford, MA, USA), at a density of 1.0 × 10^5^ cells per well (surface area: 1.7 cm^2^ per well), and incubated at 37 °C for 1 day. PLGA/Dil NPs, DOX@PLGA NPs, and DOX@PLGA/Dil NPs were added to the cells at 1 μg/ml Dil concentration and incubated for 24 h. In case of DOX@PLGA/Dil NPs + SA group, DOX@PLGA/Dil NPs were pre-incubated with SA (150 μg/ml) prior to their application to the cells. After 24 h incubation, each sample was removed and cells were washed with PBS (pH 7.4) at least thrice. Cells were immersed in 4% formaldehyde solution for fixation. Following drying the sample, VECTASHIELD mounting medium with 4′,6-diamidino-2-phenylindole (DAPI) (H-1200; Vector Laboratories, Inc., Burlingame, CA, USA) was added to each specimen for staining nuclei and inhibiting fluorescence quenching. Cellular fluorescence signals were then monitored by CLSM (LSM 880, Carl-Zeiss, Thornwood, NY, USA)^62^.

### In vitro antiproliferation test

Cytotoxicity of PHL, DOX, PLGA NPs, PLGA/PHL NPs, DOX@PLGA NPs, and DOX@PLGA/PHL NPs was tested in A549 cells by colorimetric assay^62^. A549 cells (5.0 × 10^3^ cells/well of seeding density) were loaded onto the 96-well plate and cultured at 37 °C for 1 day. PHL (1, 2.5, 5, 10, 25, 50, 100, and 200 μg/ml), DOX (0.08, 0.2, 0.4, 0.8, 2, 4, and 8 μg/ml), PLGA NPs (1, 2.5, 5, 10, 25, 50, 100, and 200 μg/ml), PLGA/PHL NPs (1, 2.5, and 5 μg/ml PHL concentration), DOX@PLGA NPs (at DOX@PLGA NPs concentrations equivalent to DOX@PLGA/PHL NPs concentrations containing 1, 2.5, and 5 μg/ml PHL), and DOX@PLGA/PHL NPs (1, 2.5, and 5 μg/ml PHL concentration) were applied to the cells and incubated for 72 h. After eliminating each specimen, CellTiter 96 AQ_ueous_ One Solution Cell Proliferation Assay Reagent (Promega Corp., Fitchburg, WI, USA) was added to the cells according to the manufacturer’s manual. The absorbance values of blank and test samples were detected at 490 nm with UV/Vis spectroscopic method (SpectraMax i3; Molecular Devices). Cell viability was calculated by dividing the absorbance value of the tested sample with that of blank (no treatment) sample^41,52,59,60,62^.

### Tumor penetration and anticancer activity studies in spheroid models

For making A549 spheroids, A549 cells were loaded to AggreWell400 24-well plate. A549 cells (2.4 × 10^5^ cells) suspended in RPMI 1640 (containing 300 mg/l L-glutamine) including heat inactivated FBS (10%, v/v) and penicillin–streptomycin (1%, v/v) were added to each well and spheroids were generated according to the manufacturer’s directions. Half of the cell culture medium was replaced with the fresh one every other day and cells were cultured for 4 days for preparing spheroids.

For the fluorescence detection, Dil-loaded NPs were prepared as described in above section and they were used in tumor penetration test. PLGA/Dil NPs, DOX@PLGA NPs, DOX@PLGA/Dil NPs, and DOX@PLGA/Dil NPs + SA (at 1 μg/ml Dil and 150 μg/ml SA concentrations) were applied to the A549 spheroids and they were incubated with orbital shaking for 24 h. In case of DOX@PLGA/Dil NPs + SA group, DOX@PLGA/Dil NPs were pre-incubated with SA (150 μg/ml) prior to the application of NPs to the spheroids. After removing each sample, A549 spheroids were rinsed with PBS (pH 7.4) at least thrice and the fluorescence signals were detected by CLSM (LSM 880, Carl-Zeiss)^21^.

For live/dead assay in A549 spheroids, approximately 40 spheroids dispersed in cell culture medium (0.2 ml) were added in each well of 12-well plate and dispersion (0.2 ml) of NPs (at 10 μg/ml PHL concentration) was applied to those spheroids. They were incubated with orbital shaking in CO_2_ incubator for 24 h. Aliquot (0.4 ml) of PBS (pH 7.4) containing calcein AM (0.2 μl) and ethidium homodimer-1 (0.8 μl) (LIVE/DEAD Viability/Cytotoxicity Kit; Molecular Probes, Inc., Eugene, OR, USA) was added to spheroids and they were incubated for 20 min in CO_2_ incubator. They were washed with PBS (pH 7.4) at least three times and their fluorescence signals were detected by CLSM (LSM 880, Carl-Zeiss).

Cell killing mechanisms in A549 spheroids after treatment of each NPs were elucidated with Reactive Oxygen Species (ROS) Detection Reagents (Molecular Probes, Inc., Eugene, OR, USA). As described in live/dead assay section, approximately 40 spheroids dispersed in cell culture medium (0.2 ml) were added in each well of 12-well plate and dispersion (0.2 ml) of NPs (at 10 μg/ml PHL concentration) was applied to those spheroids. Aliquot (0.4 ml) of PBS (pH 7.4) containing carboxy-H_2_DCFDA (10 μM) was applied to spheroids and they were incubated for 20 min in CO_2_ incubator. After rinsing with PBS (pH 7.4) at least three times, fluorescence signals from spheroids were monitored by CLSM (LSM 880, Carl-Zeiss).

### In vivo toxicity tests

In vivo toxicity of developed NPs was assessed in ICR mouse (male, 20 g of average body weight; Orient Bio, Sungnam, Korea) after intravenous administration^44,60^. The experimental procedures of the animal studies were approved by the Animal Care and Use Committee of the College of Pharmacy (Kangwon National University, Chuncheon, Korea). All animal experiments were conducted according to the National Institutes of Health guide for the care and use of laboratory animals (NIH Publications No. 8023, revised 1978). PLGA/PHL NPs, DOX@PLGA NPs, and DOX@PLGA/PHL NPs were intravenously injected at 5 mg/kg PHL dose for three times (every other day). By cardiac puncture, serum specimens were obtained from all mice. Albumin, ALT, AST, and BUN levels in serum were quantitatively determined by ALB2 (Roche Diagnostics), Alanine Aminotransferase acc. to IFCC (Roche Diagnostics), Aspartate Aminotransferase acc. to IFCC (Roche Diagnostics), and UREAL (Roche Diagnostics, Manheim, Germany), respectively, by using Cobas 8000 C702 chemical autoanalyzer (Roche Diagnostics)^44,60^. Heart, kidney, liver, lung, and spleen were also excised from the mouse. Those specimens were fixed in formaldehyde (4%, v/v) solution and fixed tissues were sliced. Those samples were then deparaffinized and hydrated with ethanol. Finally, they were stained with hematoxylin and eosin (H&E) reagent by the standard protocol^44,60^.

### Optical imaging test

For NIRF imaging test, ICG was encapsulated in PLGA NPs for their in vivo tracking following an intravenous injection. ICG (2 mg) was entrapped in PLGA NPs instead of PHL (8 mg) and same procedures in above section were applied to prepare ICG-loaded NPs. To make A549 tumor-bearing mouse models, A549 cells (at 2.0 × 10^6^ cells in 0.1 ml) were injected to the dorsal side of BALB/c nude mice (female, average body weight: 20 g, 5-weeks old; Orient Bio, Sungnam, Korea) subcutaneously^63^. Tumor volume (V, mm^3^) was calculated with the following formula: V = (1/2) × longest diameter × (shortest diameter)^2^. When the tumor volume reached 100‒200 mm^3^, dispersion of PLGA/ICG NPs or DOX@PLGA/ICG NPs (ICG dose: 2 mg/kg) was injected to the mouse via an intravenous route. Whole body signals were scanned by FOBI (NeoScience Co., Ltd., Suwon, Korea) installed with near-infrared laser filter at 0 (pre-injection), 3, and 24 h post-injection. Integrated density (= area × mean value of intensity) in the tumor region was acquired for the quantitative analysis of NIRF signals.

### Data analyses

Statistical analysis was performed with a two-tailed *t*-test and analysis of variance (ANOVA). Data are shown as the mean ± standard deviation (SD).

## Supplementary information

Supplementary Information 1.
