# Supplementary material for: Possible contribution of sialic acid to the enhanced tumor targeting efficiency of nanoparticles engineered with doxorubicin
Source: Sci Rep. 2020 Nov 12;10:19738. doi: 10.1038/s41598-020-76778-9 (PMC7661514; doi:10.1038/s41598-020-76778-9)
Supplement: Supplementary file 1 — Supplementary Information 1. [file 41598_2020_76778_MOESM1_ESM.docx]

**Supporting Information**

**Possible contribution of sialic acid to the enhanced tumor targeting efficiency of nanoparticles engineered with doxorubicin**

Song Yi Lee,^a,b,1^ Suyeong Nam,^a,1^ Ja Seong Koo,^a^ Sungyun Kim,^a^ Mingyu Yang,^a^ Da In Jeong,^a^ ChaeRim Hwang,^a^ JiHye Park,^a^ Hyun-Jong Cho^a,*^

^a^ College of Pharmacy, Kangwon National University, Chuncheon, Gangwon 24341, Republic of Korea

^b^ Kangwon Institute of Inclusive Technology, Kangwon National University, Chuncheon, Gangwon 24341, Republic of Korea

^1^These authors equally contributed to this work.

^*^Corresponding author. Tel.: +82 33 250 6916; fax: +82 33 259 5631.

*E-mail address*: hjcho@kangwon.ac.kr (H.-J. Cho)


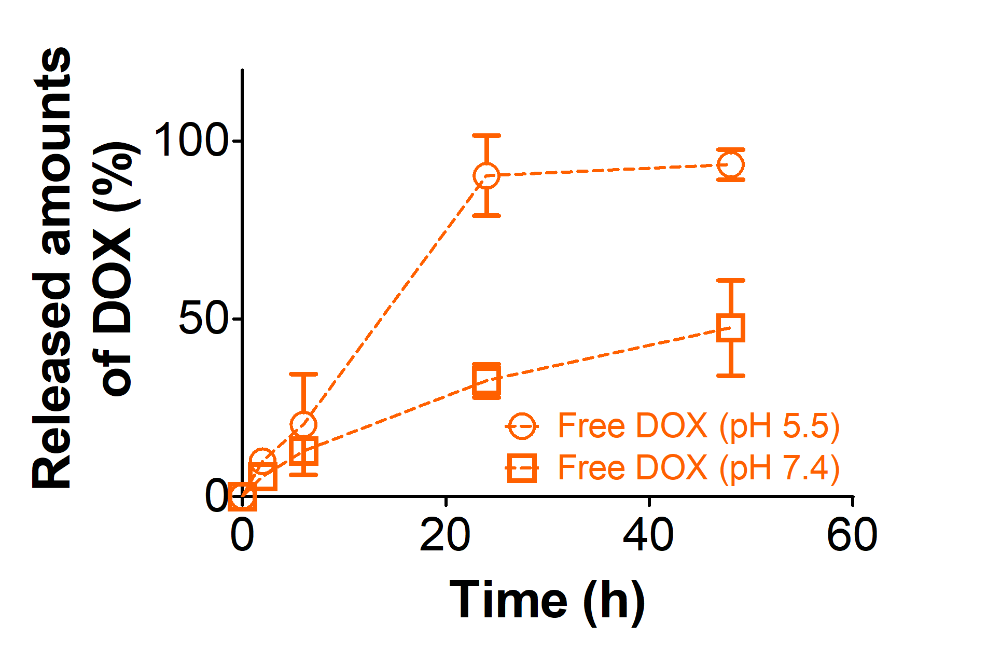


**Figure S1.** Release profile of DOX solution (free DOX) at pH 5.5 and 7.4. Each point indicates the mean ± SD (*n* = 3).


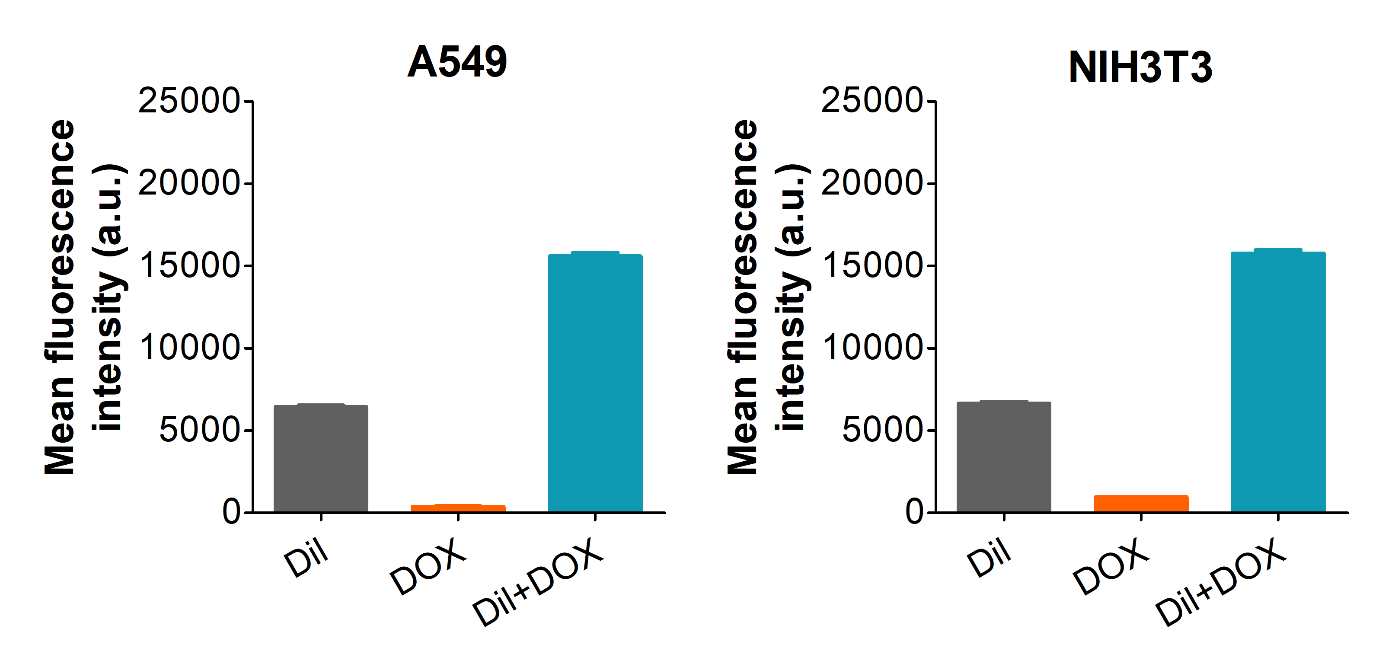


**Figure S2.** Flow cytometry data of Dil, DOX, and Dil + DOX groups in A549 and NIH3T3 cells. Mean fluorescence intensity values of each group are plotted. Each point indicates the mean ± SD (*n* = 3).
